# Minimally invasive versus conventional fixation of tracer in robot-assisted pedicle screw insertion surgery: a randomized control trial

**DOI:** 10.1186/s12891-020-03239-9

**Published:** 2020-04-06

**Authors:** Li Yongqi, Zhang Dehua, Wu Hongzi, Zhang Ke, Yang Rui, Fang Zhou, Wang Shaobo, Liao Yi

**Affiliations:** Department of Orthopedics, The Karamay Central Hospital, Karamay, 834000 China

**Keywords:** Modified, Minimally invasive, Tracer fixation, Robot, Pedicle screw placement

## Abstract

**Background:**

This study evaluated the minimal invasiveness, safety, and accuracy of robot-assisted pedicle screw placement procedure using a modified tracer fixation device.

**Methods:**

Patients were randomly assigned to conventional fixation group (25 patients) and modified fixation group (27 patients).

**Results:**

No baseline statistical difference was observed between the groups (*P* > 0.05). The length of unnecessary incision, amount of bleeding, and fixation duration for tracer fixation respectively were 6.08 ± 1.02 mm, 1.46 ± 0.84 ml, and 1.56 ± 0.32 min in the modified fixation group and 40.28 ± 8.52 mm, 12.02 ± 2.24 ml, and 5.08 ± 1.06 min in the conventional group. The difference between both groups was significant (*P* < 0.05). However, no significant difference between the two groups was observed in terms of the accuracy of pedicle screw placement (*P* > 0.05).

**Conclusions:**

The modified minimally invasive procedure for tracer fixation results in minimal trauma and is simple, reliable, and highly safe. Additionally, the procedure does not compromise the accuracy of pedicle screw placement. Thus, it has great clinical applicable value.

**Trial registration:**

**Chinese Clinical Trial Registry:** Registration number, ChiCTR1800016680; Registration Date, 15/06/2018.

## Background

Pedicle screw fixation plays an important role in reconstruction procedures for spinal stability. Given the complex morphology and variability of the pedicle, screws penetrating it may injure surrounding tissues, thus resulting in severe complications in conventional spine surgery procedures, especially those involving freehand pedicle screw insertion [1–5]. To improve the rate of successful screw placement and reduce the complications, a robotic system for spinal surgery has been recently developed. This system is highly accurate and shows good repeatability and stability, and low radiolesion and thus potentially useful in pedicle screw placement [4, 6–8].

Briefly, robot-assisted spinal operation involves the preoperative or intraoperative acquisition of images and image-guided surgery after registration and calibration [9, 10].

A tracer intraoperatively provides the origin of a coordinate system for a navigation system. A conventional tracer system includes a nonmetallic tracer and metallic spinous process clamp, which fixes the tracer on the spinous process of a patient. This tracer system is composed of a relatively stable combination and intraoperatively prevents the movement of the coordinate system origin, thereby preventing pedicle screw misplacement. An additional 3–4 cm incision is necessary for fixing the metallic clamp to adjacent spinous process, so it may lead to greater trauma, increase the risk of infection and affect fluoroscopy.

To solve the disadvantage of conventional tracer fixation, we developed a novel device for tracer fixation [11]. This device exhibited minimal invasion, simple operation, and reliable fixation. We performed a prospective randomized controlled study to compare the injury, accuracy of screw insertion, and safety of the modified tracer fixation system with those of conventional systems in robot-assisted pedicle screw insertion. We also assessed the clinical application value of the proposed system.

## Methods

### Study design

This study is a prospective, randomized, controlled trial.

### Participants

All the participants who participated in this study needed to undergo pedicle screw placement and were admitted to the Department of Orthopedics of the Karamay Central Hospital of Xin Jiang between July and December 2017. The patients suffered from thoracolumbar vertebral fracture and lumbar degenerative disease. The study was approved by The Karamay Central Hospital’s institutional review board (2018-05-23) and followed the principles of the Declaration of Helsinki of the World Medical Association. Each participant provided a written informed consent before enrollment.

The inclusion criteria were as follows: 1) > 18 years and 2) diagnosed with thoracolumbar vertebral fracture without nerve injuries (AO classification: type A or type B) [12] and lumbar vertebral degenerative disease, all having the indication of pedicle screw placement. The exclusion criteria were as follows: 1) deformed pedicles(pedicle diameters< 5 mm), 2) severe osteoporosis(t-score < − 2.5 SD), 3) comorbidities, such as serious systemic disease or coagulopathy and no tolerance to surgery, 4) poor compliance, and 5) refusal to sign the informed consent.

### General information

A total of 52 patients with pedicle screw insertion between July and December 2017 were divided into traumatic and degenerative groups. Then, each group was randomly assigned either to the conventional tracer fixation group (including 25 patients and 118 pedicle screws) or minimally invasive tracer fixation group (including 27 patients and 121 pedicle screws) through the random number table method. The patients were concealed before the randomized allocation. All the surgeries were performed by a senior doctor. Additionally, the pedicle screws were provided by Wego Medical Instruments Limited Company, and the third generation of TiRobot system came from TINAVI Medical Technologies Co., Ltd. (Beijing, China).

### Robot-assisted operation method

#### Surgical procedure of TiRobot-assisted pedicle screw insertion

The third generation of the TiRobot system is the orthopedic surgery robot independently developed in China. It consists of a robot-arm system with six degrees of freedom, infrared optical tracking system (NDI), surgical planning, and navigation system. The surgical process was divided into five phases: robot setup; collection; registration and calibration of the 3D images, parameters, and trajectory designs of the screws; robot-assisted pedicle screw placement; and verification.

The patients were under general anesthesia at the prone position. The bilateral shoulders and iliac crests were cushioned with a soft pillow to prevent abdominal compression. Before each operation, we turned on and set up the robot in a usual left unfold position. The TiRobot system, covered with a sterile plastic sheeting, was placed along the right side of the operating table, whereas the infrared optical tracking system was assembled at the head side of the patient, facing to the operating area. The patient tracer was fixed firmly on the adjacent spinous process. Three-dimensional images were acquired with a 3-D C-arm prior to calibration. The parameters and trajectory for the pedicle screw placement were set up, and the robot arm system was manually moved to the rough position of the screw insertion. Then, the robot system was run. The arm was allowed to move at the planned trajectory automatically. When the positioning accuracy indicated < 1.00 mm on the screen of the workstation, we incised the skin under the sleeve guiding, inserted the sleeve and reached the scheduled entry point, placed the guide wires, and performed screw insertion after the verification of the 3-D C-arm. We confirmed the pedicle screw position. Computed tomography (CT) scan was performed and the screw position was verified after the operation.

#### Modified minimally invasive tracer fixation device

**Fig. 1:**
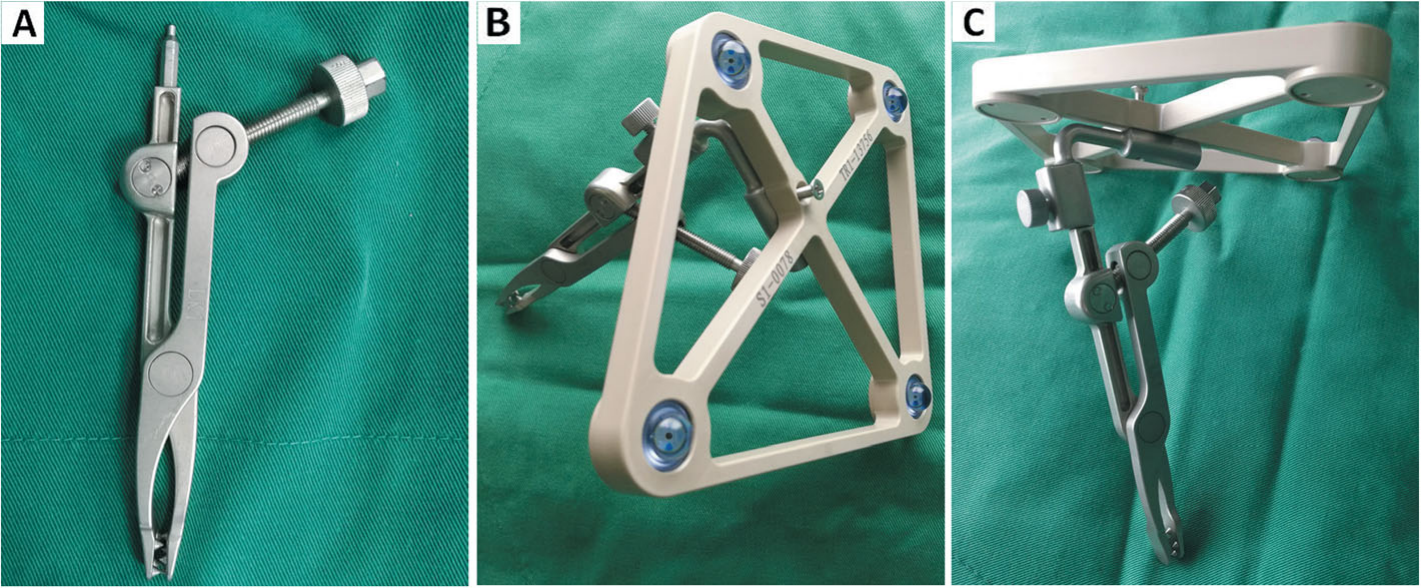
Conventional tracer fixation device: **a**. the top view of fixation device; **b**. the top view of the connection between tracer and fixation device; **c**. the bottom view of the connection between tracer and fixation device

**Fig. 2:**
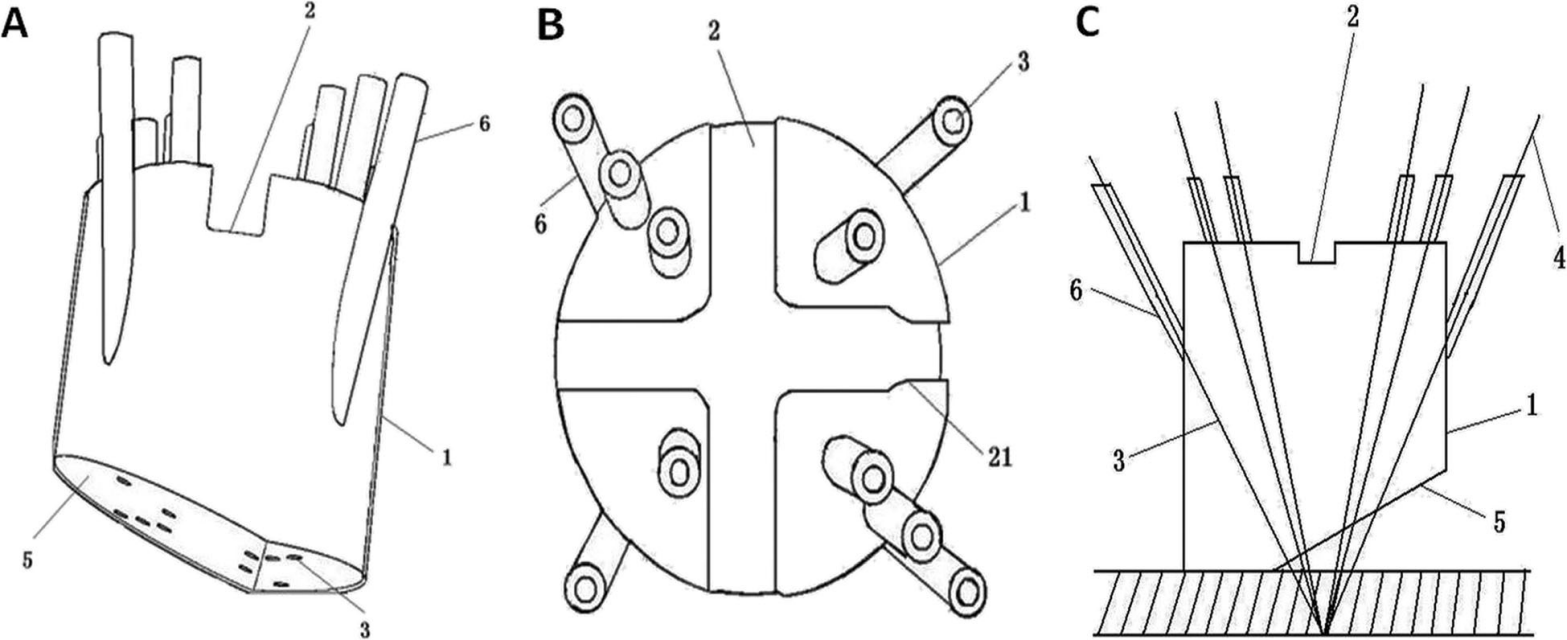
Schematic diagram of improved tracer minimally invasive fixation device: **a**. the side view; **b**. the top view; **c**. indicate the certain point the lower ends of the fixators intersect at. (1. the connector; 2. the groove; 3. the porous channel; 4. the fixator; 5. the slope; 6. the guide fixator; 21. the limitator)

**Fig. 3:**
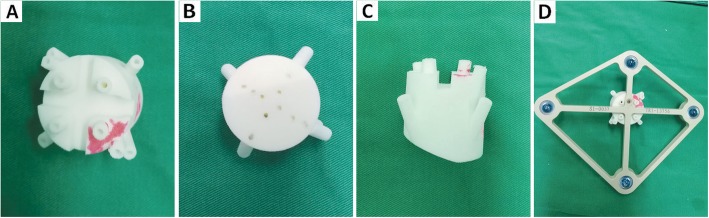
Improved minimally invasive tracer fixation device: **a**. the top view; **b**. the bottom view; **c**. the side view; **d**. the connection between tracer and improved fixation device

The conventional tracer fixation device is a metallic spinous process clamp (Fig. 1). For tracer fixation, an additional 3–4 cm incision is necessary for fixing the clamp of the device on adjacent spinous process. However, this process leads to greater trauma and may increase the risk of infection. Considering the tracer and a patients’ spinous process, we found that the modified minimally invasive tracer fixation device (Figs. 2 and 3) we developed results in simple, firm, and reliable fixation. This device which made of photosensitive resin is composed of a body, groove, porous channel, fixator, guide channel, slope of the lower surface, and limitator. A groove was employed to fix the tracer at the top of the body. Several porous channels penetrate the body from the upper to the lower surface. Moreover, the fixators used were Kirschner wires. The guide channels were situated on the top and corresponded to the porous channels. The fixators passed through channels and were fixed on the bones of the patients. To enable the slope to adapt to the physiological curve of the waist, we designed the slope beneath the surface of the body such that the coupling of the device and the skin of a patient was promoted, the impact on the pedicle screw insertion was reduced, and the connection between the tracer and NDI was facilitated.

#### Assembly of the modified minimally invasive tracer fixation device

The modified minimally invasive tracer fixation device was linked to the bottom of the support frame of the tracer. Then, place the coupling device near the spinous process of the patient and insert a Kirschner wire into the porous channel via the guide channel. Finally, the tracer system was fixed firmly on adjacent spinous process of the vertebral body to insert the screw into.

### Endpoints **and statistical methods**

The basic data were recorded and collected, including the age; sex; diagnosis; pedicle diameter; angle (e angle) between the longitudinal axis of the pedicle and sagittal axis of the vertebral body. Endpoints included primary endpoint (incision length) and secondary endpoints (amount of bleeding, duration of tracer fixation, complication involving the spinal cord and nerve root injury, development of fixation devices and the accuracy of the pedicle screw placement). For the assessment of the accuracy of the pedicle screw placement procedure, postoperative CT scans were performed on the third day after surgery, and the screw positions were evaluated separately by three independent observers: two experienced orthopedic surgeons and a radiologist who did not know the purpose of the study and assigned patients’ group. The lowest grade among the three evaluators was taken as the breach grade, so the underestimation of the breach rates of the screws was prevented. The pedicle screw positions were classified according to the Gertzbein and Robbins criteria as follows [13]: grade A, the screw is completely within the pedicle; grade B, the screw breaches the pedicle’s cortex by < 2 mm; grade C, pedicle cortical breach < 4 mm; grade D, pedicle cortical breach of < 6 mm; and grade E, pedicle cortical breach of ≥6 mm.

All statistical analyses were performed using the SPSS 21.0. The continuous variables were aggregated as the mean and standard deviation and analyzed using the independent Student t-test. Categorical variables were aggregated as the relative number and analyzed by Chi-square test. *P* < 0.05 indicates statistically significant differences for all the tests.

## Results

A total of 52 patients met the criteria for this study (conventional tracer fixation 25 vs. modified minimally invasive tracer fixation 27). The two groups had no significant differences in baseline variables, such as age, sex, diagnosis, pedicle diameter, and e angle (*P* > 0.05; Table 1).

**Table 1:** Comparison of preoperative general data between patients with conventional tracer fixation group and modified tracer minimally invasive fixation group

Group	Age (years) *M*(*Q*_*1*_,*Q*_*3*_)	Gender (Male/ female)	Diagnosis				Pedicle transverse diameter (mm) $\left(\overline{x}\pm s\right)$	e angle (^o^) $\left(\overline{x}\pm s\right)$
			Thoracolumbar fracture	Lumbar spondylolisthesis	Lumbar spondylolysis	Lumbar disc herniati-on		
C*	43 (28,56)	12/13	20	1	1	3	9.12 ± 1.22	8.82 ± 1.64
M*	41 (24,55)	13/14	21	2	1	3	8.98 ± 1.56	8.64 ± 1.48
*P* value	> 0.05	> 0.05	> 0.05				> 0.05	> 0.05

C* Conventional tracer fixation group

M* Modified tracer minimally invasive fixation group

*e angle* The angle between longitudinal axis of pedicle and sagittal axis of vertebral body

However, significant difference was observed between the modified minimally invasive tracer fixation and conventional tracer fixation groups in incision length, amount of bleeding, and duration in tracer fixation (*P* < 0.05; Table 2). The former was less than the latter.

**Table 2:** Comparison of minimal invasiveness, safety and accuracy of pedicle screw placement between patients with conventional tracer fixation group and modified tracer minimally invasive fixation group

Group	Dimension of wound (mm) $\left(\overline{x}\pm s\right)$	Amount of wound bleeding (ml) $\left(\overline{x}\pm s\right)$	Time of tracer fixation (min) $\left(\overline{x}\pm s\right)$	complication	Positions grade of screws				
					A	B	C	D	E
C*	40.28 ± 8.52	12.02 ± 2.24	5.08 ± 1.06	0	113	5	0	0	0
M*	6.08 ± 1.02	1.46 ± 0.84	1.56 ± 0.32	0	118	3	0	0	0
P value	< 0.01	< 0.01	< 0.05	–	> 0.05				

C* Conventional tracer fixation group

M* Modified tracer minimally invasive fixation group

Dimension of wound refered to the one of tracer fixation. In the group of modified tracer minimally invasive fixation, the fixators were Kirschner wire, 1.5 mm, and the size of the wound is calculated by 1.5 mm multiplying by the number of Kirschner wires. Amount of wound bleeding and time of fixation refered to the one of tracer fixation, respectively. The complication refered to spinal cord or nerve root injury when the tracer was fixed

In the modified minimally invasive tracer fixation group, 121 screws were placed, 118 of which were evaluated as Grade A; and 3, as B according to the Gertzhein and Robbins classification. Meanwhile, in the conventional tracer fixation group, 113 of the 118 screws were Grade A, and 5 were Grade B (*P* > 0.05; Table 2).

Furthermore, no complication in the spinal cord and nerve root injury were found in both groups (Table 2). During image collection and automatic registration, the Kirschner wires for tracer fixation were developed, but no image artifact displayed excellent picture quality.

## Discussion

### Characteristics and disadvantages of conventional tracer fixation device

The third generation of TiRobot is a new series connection surgical robot system, which can used by surgeons to accomplish trajectory planning based on intraoperative three-dimensional images. The robot arm system almost covers the entire spine, and intraoperative real-time navigation system ensures the accuracy and security of pedicle screw placement (Tables 1 and 2). Thus, as a burgeoning subject, using robots in surgery will be revolutionary for accurate and digital medicine [14, 15].

**Fig. 4:**
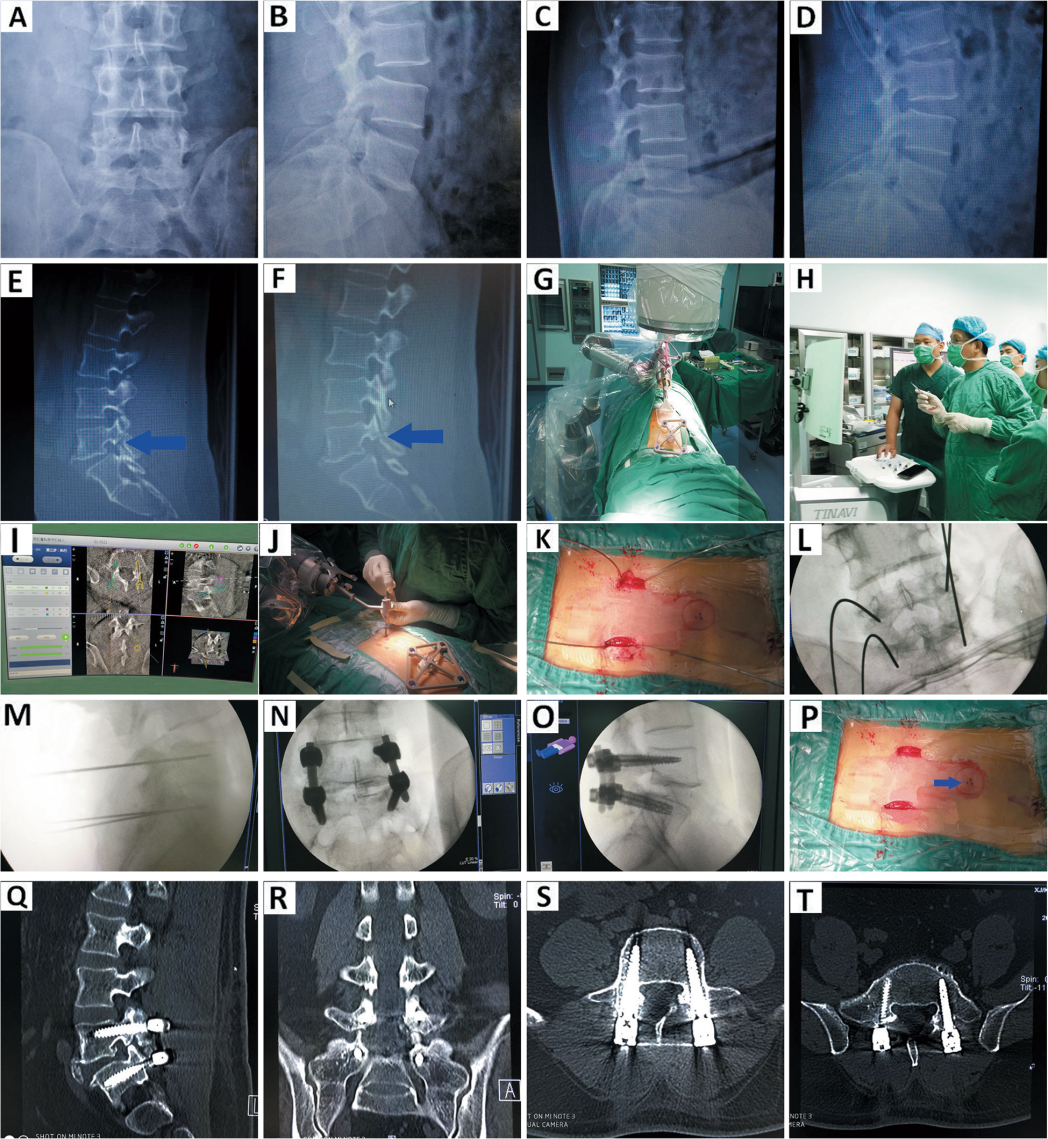
Robot-assisted pedicle screw placement and postoperative image verification with improved minimally invasive tracer fixation. The Patient was diagnosed with L5 lumbar spondylolysis and Ilumbar spondylolisthesis: **a-d**. preoperative anteroposterior, lateral and flexion-extension radiographs. **e**-**f**. preoperative sagittal CT: L5 lumbar spondylolysis and I^。^lumbar spondylolisthesis were marked with arrow(→); **g**. Three-dimensional image acquisition and registration: the method of improved tracer minimally invasive fixation was display. **i**. parameter design and path planning of pedicle screw. **j**-**k**. guide wire placement. **l**-**m**. The position verification of guide wire. **n**-**o**. Pedicle screw placement and position verification again. **p**. Postoperative wound: Minimally invasive wound was marked with arrow(→). **q**-**t**. The position grade of pedicle screws were evaluated as A via postoperative 320 CT scan

Initially, when spinal operations were performed under the guidance of the robot system, the image was obtained preoperatively from the 3D arm. Subsequently, the registration was completed prior to image-guided surgery (Fig. 4). The tracer played an important part in tracing intraoperative real-time changes in the patients’ positions and bone structures. The tracer was fastened to the bone around the surgical field and collected the corresponding intraoperative image. Finally, the image-guided surgery was accomplished through tracer positions of navigational system [16–19].

**Fig. 5:**
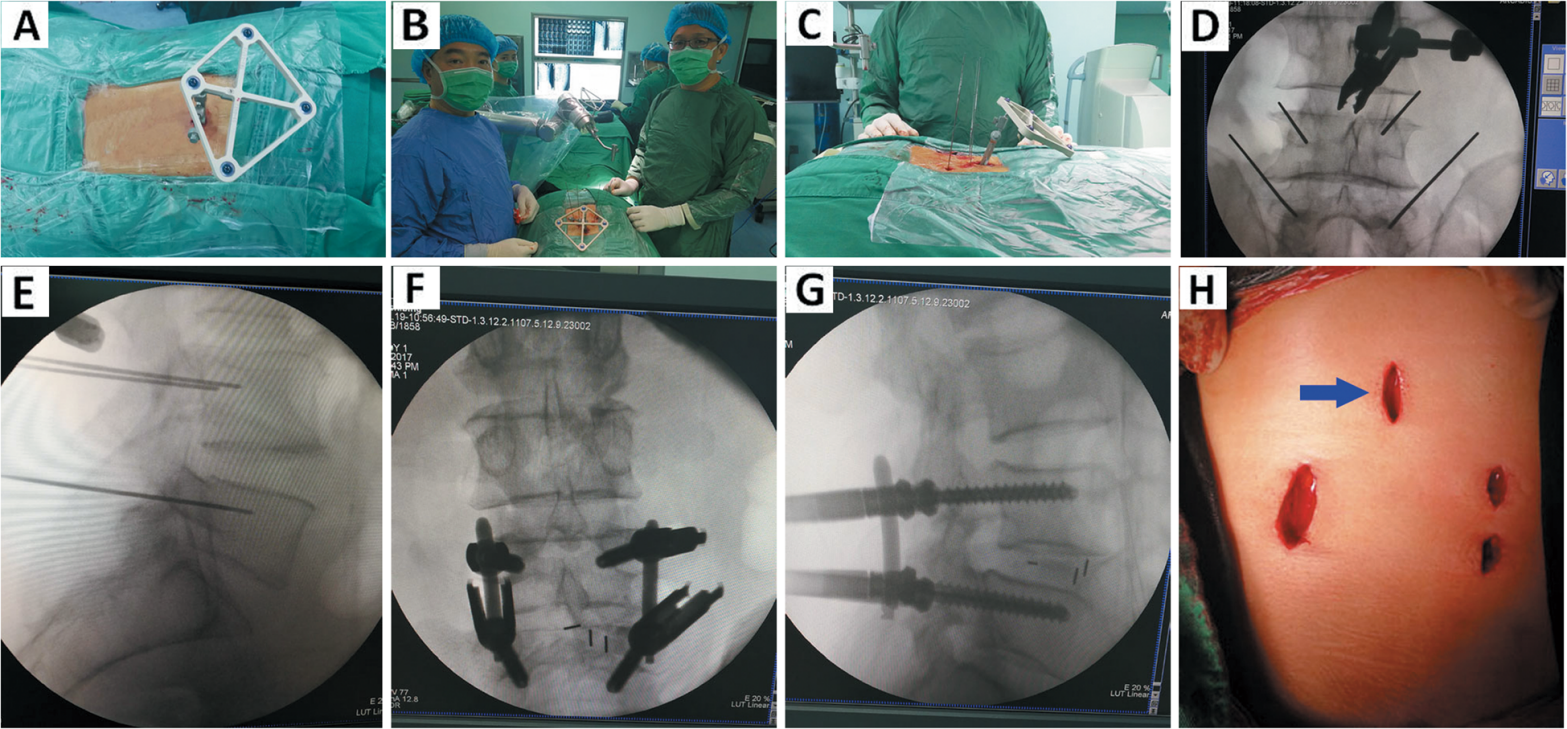
Robot-assisted pedicle screw placement and postoperative image verification with conventional tracer fixation. The Patient was diagnosed with L4 lumbar spondylolisthesis: **a**. The method of conventional tracer fixation. **c**. Robot-assisted guide wire placement. **d**-**e**. The position verification of guide wire: Metal spinous process clip was developed in fluoroscopy. **f**-**g**. Pedicle screw placement and position verification again. **h**. Postoperative wound: The wound of conventional tracer fixation was marked with arrow(→)

A conventional tracer fixation device requires an additional incision for fixing the spinous process clamp, except for the necessary ones. Thus, the use of this device leads to greater trauma and may increase the risk of infection. (Fig. 5). Furthermore, a metallic clamp has a detrimental impact on intraoperative fluoroscopy due to metal artifacts. In fact, the combination of spinous process clamp and tracer via a screw is prone to failure after long-term use. Thus, device is difficult to remove. This condition hinders the progress of the surgery and increases the risk of injury.

To solve the above technical problems, Yajun et al. [20] proposed a noninvasive tracer fixation device for spinal surgical navigation, consisting of the reference frame, pedestal, universal connecting rod, and fixing belt. When fixing the tracer, despite the no additional surgical incision, the pedestal was fastened to the back of patients with a medical adhesive, which was not firm enough. Moreover, the skin has good elasticity and mobility. Therefore, it was prone to result in tracer change in position and reduced accuracy of the pedicle screw insertion because of the soft skin and nonrigid connection between the tracer and fixation device.

### Design philosophy, advantages, and disadvantages of modified minimally invasive tracer fixation

We designed an improved minimally invasive tracer fixation device to overcome the current technical shortcomings of tracer fixation device.

One of the characteristics of this device is the axes of the channel intersect at a determined point under the modified device (Fig. 2). The fixators are fixed on the bony structure of a patient through the skin and soft tissues, which is flexible. Thus, the positions of the fixators are prone to change. However, the lower ends of the fixators intersect at a determined point and are usually fixed on the same spinous process. This condition restricts each other and prevents the device location change. For the adaptation of the device to a patient’s soft tissue, the distance required between the point and the lower surface of the connector is approximately 1.5–2.5 cm. When the patient is thin and has less subcutaneous fat, the recommended distance is 1.5 cm from the lower surface of the connector. On the contrary, when the patient is heavy and has a large amount of subcutaneous fat, 2.5 cm is suggested.

In the vertical section of the connector, the porous channels are distributed in both sides of the axis of the connector. The angles between the channels located on the same side and the axis of the connector increase in turn. This condition provides possibility that the fixators situate in different areas of the connector, and the combination tends to be fixed firmly. A slope, located on the lower surface of the device, passes through the axis of the connector to adapt to the needs of different surgical sites. For example, during lower lumbar surgery, due to the large arc of the curve, if the lower surface of the connector is flat, to fit closely with the patient’s skin is impossible, whereas if it is oblique, the close attachment will make the fixation firm. Moreover, with the slope, the tracer is inclined toward the NDI. Thus, the connection between the two is liable to be established.

The modified minimally invasive tracer fixation device has the following advantages. (1) The material is improved. The original metallic material is changed to the current photosensitive resin, which not only can be repeatedly sterilized but also has the characteristics of being safe, nontoxic, and nonallergenic. Meanwhile, the material can perform 3D printing to realize rapid production and personalized customization. (2) Nonmetallic materials are able to effectively avoid image interference from metal artifacts of the fixation device in fluoroscopy. (3) The Kirschner wires are used to fix the base and the tracer, reducing the injury and infection risk. (4) The device without screw fixation effectively avoids the screw failure on spinous process clamp. This phenomenon will cause difficulty in removing the clamp from the spinous process.

The main drawback of this fixation method is that the fixators use the Kirschner wire without the limiter. The depth of the inserted Kirschner wire mainly depends on the experience of surgeon, and the Kirschner wire can possibly enter the spinal canal, leading to serious consequences. Therefore, adding the limiter to the fixator to avoid the risk of nerve injury is necessary.

### Minimal invasiveness, accuracy, feasibility, and safety analysis of the modified minimally invasive tracer fixation device

**Fig. 6:**
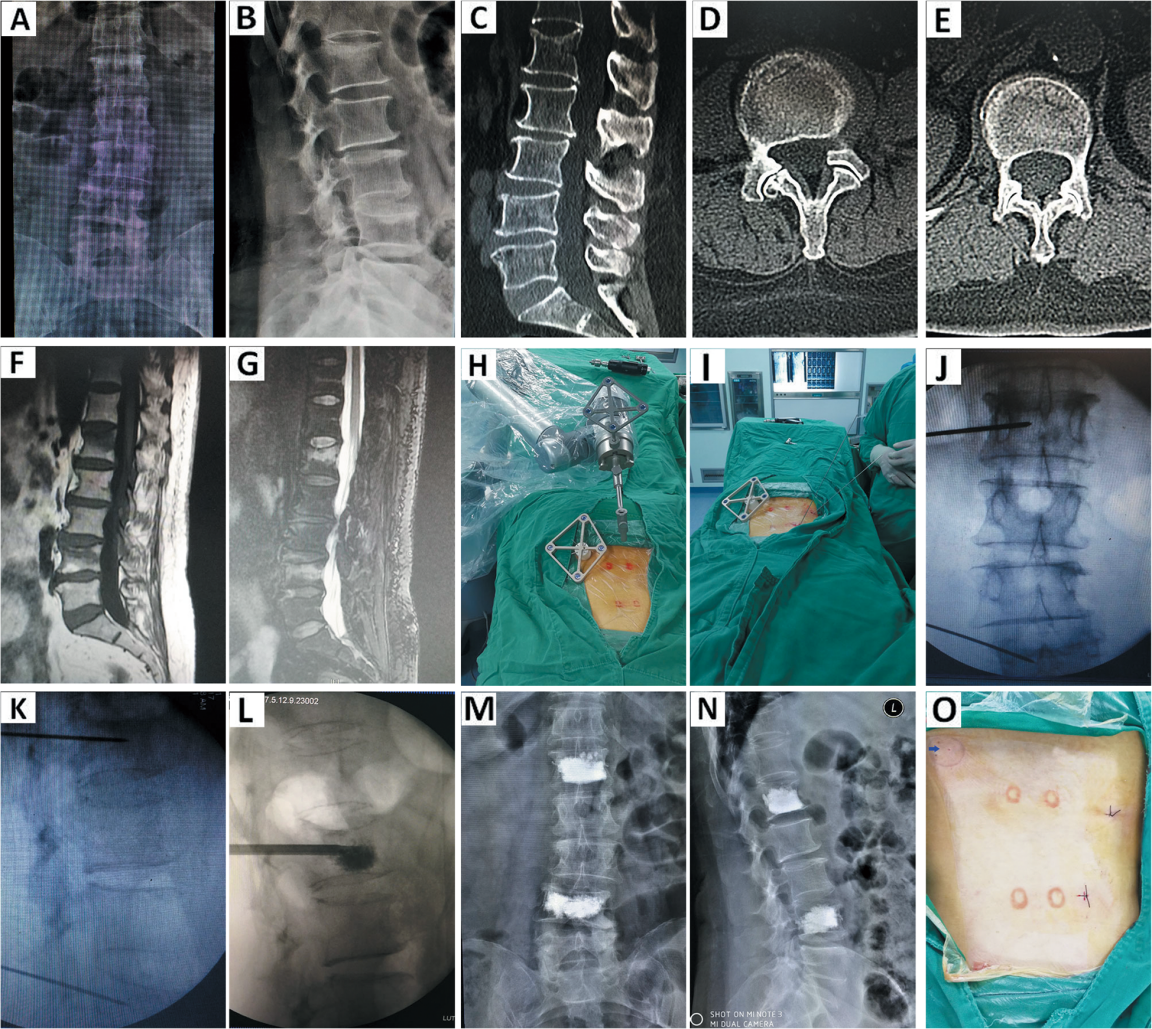
Robot-assisted PVP or PKP and postoperative image verification with improved minimally invasive tracer fixation. The Patient was diagnosed with lumbar osteoporotic fracture(L1 and L4): **a**-**b**. preoperative anteroposterior and lateral radiographs. **c**-**e**. preoperative sagittal and axial CT. **f**-**g**. Preoperative sagittal MRI showed abnormal signal of bone marrow edema(L1 and L4). **h**. The method of improved minimally invasive tracer fixation. **i**. Robot-assisted guide wire placement. **j**-**k**. The position verification of guide wire. **l**. Bone cement injection under fluoroscopy. **m**-**n**. Postoperative image verification. **o**. Postoperative wound: Minimally invasive wound was marked with arrow(→)

This study included 52 patients. No significant difference was observed between the minimally invasive and conventional tracer fixation procedures in the baseline variables, such as age, sex, diagnosis, pedicle diameter, and e angle. Compared with the conventional group, minimally invasive tracer fixation group did not suffer from unnecessary surgical wound, showed less bleeding, presented statistically significant differences. Thus, the minimally invasive tracer fixation method accorded with the philosophy of contemporary minimally invasive surgery particularly by further reducing the risk of injury and infection. Furthermore, this method can relieve post-operative pain and shorten hospital stays. This minimal advancement promotes further expansion of indication of robot-assisted minimally invasive surgery, such as robot-assisted percutaneous vertebroplasty/percutaneous kyphoplasty (PVP/PKP), or robot-assisted operation will be meaningless for PVP/PKP surgery, as shown in Fig. 6. Additionally, this device has been successfully used to minimally invasive surgery of the upper cervical spine and other parts of the body suitable for robot-assisted surgery in our department.

No significant differences were observed in the accuracy of pedicle screw placement (*P* > 0.05) between both groups. This result revealed that the modified minimally invasive tracer fixation device does not compromise accuracy during pedicle screw placement compared with conventional fixation and inherits the natural advantage of high accuracy in robot-assisted spine surgery. The device is highly clinically feasible with the characteristics of short tracker fixation duration, simple, and reliable fixation method. Furthermore, no image artifact displayed excellent picture quality despite the Kirschner wire development during image collection and automatic registration. In fact, no complications of the spinal cord and nerve root injury were found for both groups in the intraoperative and postoperative periods. This result demonstrates that the device is highly safe and relieves the concerns of patients, surgeons, and robotic engineers on severe complications of spinal surgery. Moreover, this finding promotes the pervasive application of the device in robot-assisted pedicle screw placement surgery.

## Conclusions

The minimal invasiveness, accuracy, and intelligence of surgery are the trend landmark of the development of surgical robot. As an important link to reflect the minimally invasive and accurate robot, fixation method of the tracer is of great significance during robot-assisted pedicle screw placement. The modified minimally invasive tracer fixation device presented the advantage of minimal invasiveness, firm fixation, simple, and reliable operation. Meanwhile, this fixation had no influence on the accuracy and safety of robot-assisted pedicle screw insertion. Thus, its great value in clinical application is proven. However, this study is a single-center study, and the sample size is relatively low. It is necessary for multi-center, large-sample studies to further explore.

## Data Availability

The datasets used and/or analysed during the current study are available from the corresponding author on reasonable request.

## Abbreviations

NDI: Infrared optical tracking system
CT: Computed tomography scan
PVP/PKP: Percutaneous vertebroplasty/percutaneous kyphoplasty
